# Negative effects of time autonomy in digital collaboration

**DOI:** 10.1007/s11612-023-00671-y

**Published:** 2023-02-21

**Authors:** Rebekka Mander, Conny H. Antoni

**Affiliations:** Work and Organizational Psychology, FB I, Trier University, 54286 Trier, Germany

**Keywords:** Emotional Exhaustion, Job-to-home spillover, Time-pressure, Digital collaboration, Job demands, Emotionale Erschöpfung, Entgrenzung, Zeitdruck, Digitale Kollaboration, Arbeitsanforderungen

## Abstract

This article in the journal *Group. Interaction. Organization (GIO) *focuses on the effects of time autonomy on job-to-home spillover and emotional exhaustion when employees face high time pressure in digital collaboration. We assume time autonomy to strengthen rather than to buffer the detrimental indirect effects of high time pressure on emotional exhaustion via job-to-home spillover, and that high digital collaboration amplifies this effect even further. In a sample of *N* = 111 employees, we investigated this research question using conditional process analysis. Results support our assumptions as for high digital collaboration high time autonomy amplified the detrimental indirect effect of time pressure on emotional exhaustion via job-to-home spillover, whereas for low digital collaboration high time autonomy buffered this indirect effect. Thus, employees’ risk for emotional exhaustion is higher when time pressure, time autonomy and digital collaboration are high. Therefore, employees and leaders should carefully consider their choices concerning the use of time in the context of digital collaboration.

**Supplementary Information:**

The online version of this article (10.1007/s11612-023-00671-y) contains supplementary material, which is available to authorized users.

## Abstract (de)

Dieser Artikel in der Zeitschrift Group. Interaction. Organization (GIO) befasst sich mit den Wirkungen der Zeitautonomie auf das Übergreifen der Arbeit auf das Privatleben und die emotionale Erschöpfung, wenn Mitarbeiter bei digitaler Zusammenarbeit unter hohem Zeitdruck stehen. Wir gehen davon aus, dass Zeitautonomie die schädlichen indirekten Auswirkungen von hohem Zeitdruck auf die emotionale Erschöpfung durch das Übergreifen von der Arbeit auf das Privatleben eher verstärkt als abpuffert, und dass eine hohe digitale Zusammenarbeit diesen Effekt noch weiter verstärkt. In einer Stichprobe von N = 111 Arbeitnehmern haben wir diese Forschungsfrage mit Hilfe der bedingten Prozessanalyse untersucht. Die Ergebnisse stützen unsere Annahmen, da bei hoher digitaler Zusammenarbeit eine hohe Zeitautonomie den schädlichen indirekten Effekt von Zeitdruck auf die emotionale Erschöpfung durch das Übergreifen der Arbeit auf das Privatleben verstärkt, während bei niedriger digitaler Zusammenarbeit eine hohe Zeitautonomie diesen indirekten Effekt abpuffert. Somit ist das Risiko der emotionalen Erschöpfung bei Arbeitnehmern höher, wenn Zeitdruck, Zeitautonomie und digitale Zusammenarbeit hoch sind. Daher sollten Mitarbeiter und Führungskräfte ihre Entscheidungen bezüglich der Zeitverwendung im Rahmen der digitalen Zusammenarbeit sorgfältig abwägen.

## Changing working conditions in the Covid-19 pandemic

Modern working arrangements are becoming more and more flexible in terms of working hours and location, reinforcing the technological trend for team members and leaders to increasingly collaborate virtually. While this shift in work patterns inherent in work 4.0 is beneficial in many ways, it also places a variety of demands on employees (Schaff 2019). Virtual work can become a demand when combined with time pressure (Wang et al. 2021) leading to emotional exhaustion (in short exhaustion) in the long run (De Beer et al. 2016). In virtual collaboration higher flexibility comes with higher demands for self-organization (Kauffeld et al. 2022). Autonomy is known to be a job resource (Demerouti and Nachreiner 2019) and has benefits like reduced work-home-conflict (Allen et al. 2013). However, current studies suggest, that autonomy can have ambivalent effects through mediating and moderating variables. For example, Bredehöft et al. (2015) and Dettmers and Bredehöft (2020) report that autonomy can have ambivalent effects due to high job design and self-organization demands (namely planning demands, demands to regulate effort and development demands) in modern work structures.

With the beginning of the Covid-19 pandemic, working conditions changed in that time autonomy, and remote digital work were largely expanded. Employees experienced stronger conflicts of work and home than before (Oksanen et al. 2021). The blurring of borders between work and home is a risk that can lead to job-to-home spillover (in short spillover), the burdening feeling of work interfering with private life. When work and private life compete for time, which is a limited resource, and employees have to decide where to invest their scarce time, time autonomy may be a problem for employees. If employees face high time pressure, they are more likely to choose to work longer hours to get their work finished (Lott 2018). Thus, higher levels of time autonomy may increase the positive relation between time pressure and spillover. As spillover can cause conflicts in private life and reduce social resources it may lead to stress, exhaustion and impaired well-being (Lee et al. 2016). Therefore, we assume that spillover is a mediating mechanism explaining the effects of time pressure on exhaustion that is amplified by high time autonomy.

Spillover might further increase if people with high autonomy must collaborate digitally. When collaboration is digital, it is harder to achieve a trustful working environment than in face-to-face working environments (Breuer et al. 2016). Digital collaboration can also be more challenging than face-to-face in terms of effectivity and scheduling (Wang et al. 2021). Job design demands might therefore increase as more challenges sum up. In combination with high time autonomy this can strengthen the occurrence of job-to-home spillover, as borders of job and private life blur and one can be contacted any time anywhere when collaborating digitally. Extended availability may occur impairing mental health (Menz et al. 2016). This combination of high time pressure, time autonomy and digital collaboration may contribute to a highly demanding working scenario increasing the risk of spillover and exhaustion. Empirical evidence for these considerations is still scarce. Therefore, we aim to contribute to the literature in three ways. First, by analyzing spillover as a potential mediating mechanism in the relationship between time pressure and emotional exhaustion. Second, by clarifying the role of time autonomy, and third, of digital collaboration as crucial working conditions influencing this potential mediating mechanism.

## The indirect effect of time pressure on emotional exhaustion via job-to-home spillover

Time pressure is defined as the lack of time to complete job-related tasks (Schmitt et al. 2015). Time pressure can come in various forms. It can derive from goals that are set by others or by oneself and that cannot be managed in the available time. It can also stem from last-minute requirements or changes of requirements that make it difficult to meet the deadline. According to the job demands-resources (JD-R) model, time pressure is a job demand, which consumes energy and increases exhaustion, particularly if resources are low (Bakker and Demerouti 2017). Exhaustion is a sub dimension of burnout and is widely used to measure mental stress. Exhaustion refers to the feeling of being emotionally overworked and drained by work-related contact with people (Demerouti and Nachreiner 2019). It is the consequence of working until feeling depleted of energy (Schaufeli et al. 1996) or of engaging in self-endangering behavior (Baeriswyl et al. 2018).

With high job demands, such as time pressure, in modern work arrangements, it is important to establish resources in order to maintain psychological well-being. When work and private life mix, this can become more difficult. The blending of work and private life is known as dissolution of boundaries, which has both advantages and disadvantages, as flexibility is gained through the blending of the life domains. On the one hand, it can facilitate positive processes, such as using the flexibility for personal matters (e.g., a parent may interrupt work for an hour to pick up their child); on the other hand, conflicts can arise between the two life domains (Allen et al. 2013). Conflict between the two life domains can lead to higher experienced strain (Byron 2005). Often, a lack of time is the underlying problem in balancing work and private or family life (Falter Mennino et al. 2005; Lott 2018). High time pressure negatively impacts leisure time causing spillover, the negatively connoted form of dissolution of boundaries, in which the job spills over into the private life (Falter Mennino et al. 2005). According to Kossek (2016), health is at risk when unintentional blending of boundaries occurs. As spillover can lead to conflicts in private life, reduced leisure time and social resources, it can be associated with exhaustion and impaired well-being (Lee et al. 2016). As research shows that time pressure can lead to spillover and that both can enhance exhaustion, we assume that time pressure enhances exhaustion through increasing spillover:

### H1:

*Time pressure increases exhaustion indirectly through spillover*.

## Interaction of time pressure, time autonomy, and digital collaboration

We stated earlier that modern working arrangements pose new job design demands. In this study, we are interested how time pressure, time autonomy and digital collaboration interact and influence spillover and exhaustion. Time autonomy describes the degree to which employees can decide when they work and how they structure their workday. Employees who have the freedom to decide over their worktime, are challenged to respond to self-organization requirements (Dettmers and Bredehöft 2020). When there are no or few external constraints, one must be careful to progress at a reasonable pace without losing sight of relaxation (Bredehöft et al. 2015). Working longer hours, skipping breaks, or working even though one feels sick are self-endangering behaviors, and risks for employees with high levels of autonomy. They are associated with emotional exhaustion (Baeriswyl et al. 2018). Self-discipline to focus on work and stick to one’s time schedule can be challenging when you work remotely and digitally and are more easily distracted (Wang et al. 2021). This is to say, that time autonomy can be a challenge, that makes coping with high time pressure even harder. Under high time pressure, high levels of time autonomy can lead to increased spillover, as employees may choose to work longer hours to get their work done (Lott 2018) and can thus amplify the indirect effect of time pressure on exhaustion through spillover. Thus, we propose:

### H2:

*Time autonomy moderates the effect of time pressure on job-to-home spillover, such that the effect is stronger for high than for low time autonomy (H2a) and that high time autonomy amplifies the indirect effect of time pressure on exhaustion via job-to-home spillover (H2b)*.

Digital collaboration describes the degree to which employees must collaborate with others via digital media to get their work done. If people must collaborate digitally and remotely, as it was the case during the Covid-19 pandemic, negative spillover might further increase, as competing demands of work and non-work environments may become more salient (Wang et al. 2021). Data on the impact of the Covid-19 pandemic on working life support this reasoning as they indicate that collaboration via digital media increased, and employees worked longer hours per day compared to pre-pandemic times (DeFilippis et al. 2020). Digital collaboration is related to information overload (Korunovska and Spiekermann 2019) that can lead to emotional exhaustion (Oksanen et al. 2021). Further, digital collaboration can make work more complex by introducing new tools and processes and therefore increase self-organization demands (Hardwig and Weißmann 2021). Digital collaboration can increase demands, given the beforementioned circumstances. Therefore, we suggest that when people both have time autonomy and collaborate digitally the effect of time pressure on job-to-home spillover and the indirect effect of time pressure on exhaustion is further amplified:

### H3:

*Digital collaboration amplifies the moderating effect of time autonomy on the association between time pressure and job-to-home spillover (H3a), and the indirect effect of time pressure on exhaustion via job-to-home spillover (H3b), so that it is stronger for high digital collaboration and high time autonomy compared to low digital collaboration digital collaboration and high time autonomy.*

**Fig. 1:**
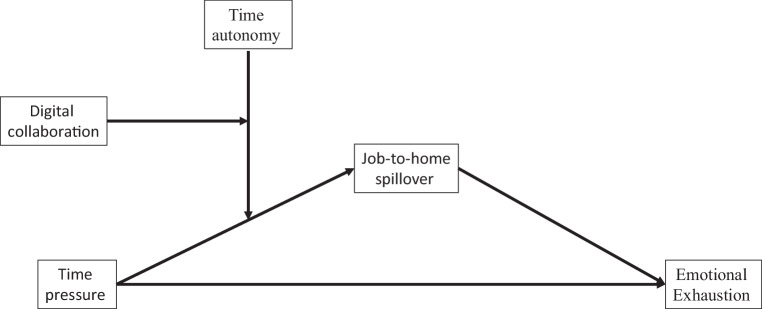
Research Model

Our theoretical considerations are visualized in Fig. 1.

## Method

### Sample

The study sample was recruited via an online link for employees who were enrolled at a university of applied science in addition to working in their job and had agreed to participate in this study. 118 persons responded. 111 data sets could be used for the analysis. Seven data sets had to be excluded due to duplicate data as some respondents responded to the survey twice. Almost all participants (96%) were studying part-time. Respondents’ average age was 29 years (*SD* = 6.6) ranging from 21 to 52 years. 75% of the respondents were women and 25% were men. On average, 87% of the respondents worked using digital media and 62% of the time they collaborated digitally with others. On average, respondents worked almost two days per week from home (*M* = 15.8 h; *SD* = 17.7), compared to five hours before the start of the Covid-19 pandemic. 18% of the respondents held leadership positions. Actual working hours averaged 34.7 h, while contracted working hours averaged 33.1 h.

### Measures

All variables except digital collaboration were measured using a five-point Likert scale ranging from “strongly disagree” to “strongly agree” with good internal consistencies (0.88 < Cronbach’s α < 0.92, cf. Table 1).

**Table 1:** Descriptive Statistics, Correlations, and Internal Consistencies of Study Variables

Measure	*M*	*SD*	1	2	3	4	5
1. Time pressure	2.91	0.98	*0.89*	*–*	–	–	–
2. Job-to-home spillover	2.76	0.98	0.49**	*0.90*	*–*	–	–
3. Time autonomy	3.80	0.86	−0.05	−0.37**	*0.88*	–	–
4. Digital collaboration	61.80	32.21	0.17^+^	−0.07	0.18^+^	*–*	*–*
5. Emotional exhaustion	2.86	1.03	0.42**	0.59**	−0.28**	−0.13	*0.92*

*Note. N*  = 111. The internal consistencies Cronbach’s alphas are depicted along the diagonal in *italics*

^+^ *p* < 0.1 * p < 0.05 ** *p* < 0.01 (two-tailed)

#### Time pressure

Time pressure was measured by five items. Four items were adapted from the Instrument for Stress-Oriented Task Analysis (ISTA; Irmer et al. 2019; e.g., “I often have time pressure?”) One item was taken from the Kurz-Fragebogen zur Arbeitsanalyse (KFZA; Prümper et al. 1995; “I have too much to do”).

#### Time autonomy

Time autonomy was measured using five items. Three items refer to scheduling autonomy (Breaugh 1999). For example, “I have a say in when I do what at work.” Two items refer to time related aspects of decision autonomy (Chevalier and Kaluza 2015). An example item is “I can decide for myself when to take a break.”

#### Digital collaboration

Digital collaboration was measured with a single-item. Participants were asked to rate how often they collaborate with other persons via digital media from never (0%) to always (100%).

#### Job-to-Home spillover

Spillover was measured using five items from Falter Mennino et al.’s (2005) scale (e.g., “I was not in a good mood when I was home because of my work.”). We changed the time frame to four weeks and the corresponding frequency rating of the original scale (“In the past three months, how often …”) as the study was done at the beginning of the pandemic when disrupt changes in digital collaboration overwhelmed organizations and we wanted to refer to this context and not to mix current experiences with what happened before.

#### Emotional exhaustion

Exhaustion was measured with six items. Five items from the Maslach Burnout Inventory—General Survey (MBI-GS) (Schaufeli et al. 1996) using the German version (Cillien et al. 2006) and one additional item (“I will not be able to continue doing this job indefinitely.”) which was also used by Gerlmaier and Latniak (2011) to include the notion of constrained work ability in the long run (Ilmarinen 2009).

## Results

The hypotheses were tested using SPSS 27 and model 4 and 11 of the macro PROCESS (Hayes 2018). PROCESS is used for testing mediation, moderation, and conditional process analysis. We used bias-corrected bootstrapping with 10,000 replicate samples in order to account for the non-normal distribution of outcome variables. Given the relatively low sample size for testing complex interaction effects with low effect sizes, a 90% confidence interval [CI] was applied (the lower level [LL] and upper level [UL] of the confidence interval will be specified). Significance of effects is indicated when confidence intervals do not include zero.

Descriptive statistics and intercorrelations are displayed in Table 1. Intercorrelations between job to home spillover, exhaustion and time pressure were positive, and with time pressure negative. Thus, employees perceiving higher time pressure are also reporting higher spillover and exhaustion, and employees perceiving higher time autonomy are reporting lower spillover and exhaustion, similar to prior research findings described above.

### Hypothesis testing

We assumed that time pressure increases exhaustion indirectly through spillover (H1). Hypothesis 1 was tested using Model 4 of the PROCESS macro (Hayes 2018). In line with Hypothesis 1 results indicated a positive indirect effect of time pressure on exhaustion through job-to-home spillover (*B* = 0.26, *SE* = 0.10, LL = 0.1194, UL = 0.4437, *R*^*2*^ = 0.37, *p* < 0.001). However, results indicated also a positive direct effect of time pressure on exhaustion (*B* = 0.18, *SE* = 0.09, LL = 0.0236, UL = 0.3289). This means that increasing time pressure may lead both directly and indirectly through job-to-home spillover to more exhaustion. Detailed results are displayed in the attached supplemental tables.

Hypothesis 2 states that time autonomy amplifies the effect of time pressure on job-to-home spillover (H2a) and the indirect effect of time pressure on exhaustion via job-to-home spillover (H2b) The hypotheses were tested using Model 7 of the PRCOESS macro (Hayes 2018). Results did neither indicate a significant interaction effect of time pressure and time autonomy on job-to-home spillover (*B* = 0.11, *SE* = 0.08,* △R*^*2*^ = 0.01, *p* = 0.18), nor a moderated indirect effect of time pressure via job-to-home spillover on exhaustion by time autonomy (Index = 0.06; SE = 0.06, LL = −0.0818, UL = 0.1295). Therefore, both Hypotheses 2a and 2b had to be rejected. Only time autonomy (*B* = −0.70, *SE* = 0.24, *p* < 0.01, *R*^*2*^ = 0.38, *p* = 0.01) was negatively related to spillover, and both time pressure (*B* = 0.18, *SE* = 0.09, *p* < 0.05) and spillover positively to exhaustion (*B* = 0.54, *SE* = 0.09, *p* < 0.01, *R*^*2*^ = 0.38, *p* = 0.01). However, the positive sign of the interaction effect and the indirect effect coefficients of time pressure via job-to-home spillover on exhaustion from low (*B* = 0.20, *SE* = 0.13, LL = 0.0647, UL = 0.4685) to high time autonomy (*B* = 0.30, *SE* = 0.08, LL = 0.1763, UL = 0.4446) at least indicate associations in the expected direction. Nevertheless, given the non-significant interaction effect results do not support that higher time autonomy amplifies the effect of time pressure on job-to-home spillover (H2a) and the indirect effect of time pressure on exhaustion via job-to-home spillover (H2b).

Hypothesis 3a stated that digital collaboration amplifies the moderating effect of time autonomy on the effect of time pressure on job-to-home spillover (H3a), and the indirect effect of time pressure on exhaustion via job-to-home spillover (H3b), so that it is stronger for high digital collaboration and high time autonomy compared to low digital collaboration and high time autonomy.

These hypotheses were tested using Model 11 of the PRCOESS macro (Hayes 2018). Results indicated significant main and interaction effects of time pressure, time autonomy, and digital collaboration on job-to-home spillover (*R*^*2*^ = 0.44, *p* < 0.01). The proposed three-way interaction was significant (*B* = 0.01, *SE* = 0.003, LL = 0.0046, UL = 0.0157, *△R*^*2*^ = 0.05, *p* < 0.01, cf. ESM 1) supporting hypothesis 3a. A closer look indicates disordinal moderated interaction effects with time autonomy amplifying the association between time pressure and spillover for high digital collaboration (*B* = 0.37, *p* < 0.01), and buffering it for low digital collaboration (*B* = −0.26, *p* = 0.07), and not changing it for medium digital collaboration (*B* = 0.05, *p* = 0.52).

**Fig. 2:**
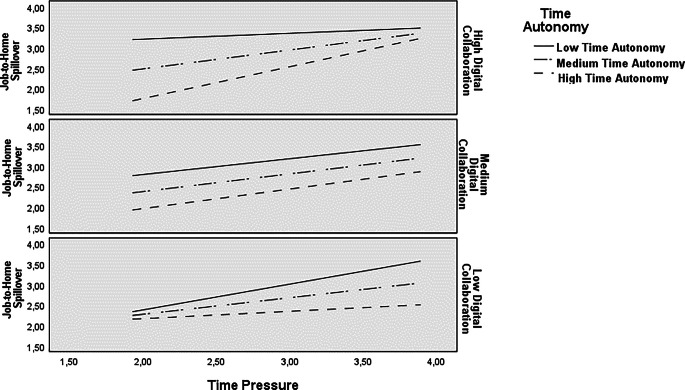
Three-Way Interaction Between Time Pressure, Time Autonomy and Digital Collaboration on Job-to-Home Spillover

Figure 2 displays the three-way interaction effects in detail.

As expected in hypothesis 3a, time pressure was most strongly related to spillover when both time autonomy and digital collaboration were high, indicating their assumed amplifying effect (*B* = 0.78, *SE* = 0.13, LL = 0.5534, UL = 0.0014, cf. ESM 2). When time autonomy was high and digital collaboration was low time pressure was not associated with spillover, that is, even when time pressure increased spillover remained almost unchanged, indicating a buffering effect under these conditions. There was also no significant relationship between time pressure and spillover when time autonomy was low and digital collaboration high. However, time pressure was strongly related to spillover when both time autonomy and digital collaboration were low (*B* = 0.63, *SE* = 0.17, LL = 0.33811, UL = 0.9220), indicating that increasing time pressure goes along with increasing spillover under these conditions.

Hypothesis 3b stated that the indirect effect of time pressure through job-to-home spillover on exhaustion will be strengthened for those who report high time autonomy and collaborate digitally to a high extent.

The indirect effects of time pressure on exhaustion via job-to-home spillover moderated by time autonomy by digital collaboration were significant (index of moderated moderated mediation: *B* = 0.005, *SE* = 0.003, LL = 0.0015, UL = 0.0105; cf. Table 2). Supporting Hypothesis 3b, the moderated indirect effect of time pressure on exhaustion by time autonomy was positive and strongest for high digital collaboration (*B* = 0.19, LL = 0.0351, UL = 0.3586), indicating that high digital collaboration amplifies the moderated indirect effect of time pressure on exhaustion by time autonomy. For low digital collaboration the moderated indirect effect of time pressure on exhaustion by time autonomy was negative but not significant (*B* = −0.14, LL = −0.3508, UL = 0.0023), indicating a non-significant buffering effect.

**Table 2:** Indices of Moderated Mediation Analysis for Time Pressure on Emotional Exhaustion via Job-to-Home Spillover by Time Autonomy by Digital Collaboration

	Indices of conditional moderated mediation	*SE*	90% CI	
Values of moderator (−1 *SD*. *M*. +1 *SD*)			LL	UL
Emotional exhaustion				
Low digital collaboration (30.59)	−0.1405	0.1112	−0.3508	0.0023
Medium digital collaboration (61.80)	0.0284	0.0592	−0.0924	0.1029
High digital collaboration (93.01)	0.1973	0.0982	0.0351	0.3586
Index of moderated moderated mediation	0.0054	0.0028	0.0015	0.0105

Note. *N* = 111; *bootstrap sample size* = 10.000

*90% CI* confidence interval, *LL* lower limit, *UL* upper limit

## Discussion

The aim of this study was to extend the existing state of research by analyzing spillover as a potential mediating mechanism in the relationship between time pressure and emotional exhaustion, and by clarifying the role of time autonomy and digital collaboration as crucial working conditions influencing this potential mediating mechanism. We hypothesized time autonomy to strengthen the detrimental indirect effect of time pressure on exhaustion via spillover, when digital collaboration is high rather than low.

### Theoretical implications: interaction of time pressure, time autonomy and digital collaboration

In Hypothesis 1 we proposed an indirect effect of time pressure on exhaustion through spillover. Results support this hypothesis and our reasoning that increasing time pressure is associated with job demands that spill over into private life (Falter Mennino et al. 2005), conflicts in private life, reduced leisure time and social resources, and increasing exhaustion (Lee et al. 2016). However, this seems not to be the only mechanism for increasing exhaustion, as controlling for spillover, we found also a direct path between time pressure and exhaustion. This might indicate that time pressure leads to exhaustion because it depletes one’s resources when one is constantly trying to achieve goals without having enough time (Demerouti and Nachreiner 2019).

In Hypotheses 2 we assumed that high time autonomy amplifies the effect of time pressure on job-to-home spillover (H2a) and through spillover indirectly on exhaustion (H2a). Although the positive sign of the interaction effect and the indirect effect coefficients of time pressure via job-to-home spillover on exhaustion from low to high time autonomy showed results in the expected direction, we did not find the expected significant moderating effects to support Hypotheses 2a and 2b. One reason might be that the sample size was too small for testing such a small interaction effect size.

In Hypotheses 3 we assumed that when employees collaborate digitally to a high extent this amplifies the moderating effect of time autonomy on the effect of time pressure on job-to-home spillover (H3a) and thus also the indirect effect of time pressure on exhaustion via job-to-home spillover. Results indicated support for this assumed amplifying effect of digital collaboration. Also, the indirect association of time pressure via job-to-home spillover on exhaustion was positive and strongest when both time autonomy and digital collaboration were high, supporting H3b. However, we found also support for the buffering effects of digital collaboration, as time pressure was not associated with spillover when time autonomy was high and digital collaboration low. Also, there was no significant moderated indirect effect of time pressure on exhaustion by time autonomy for low digital collaboration. These findings contribute to research by clarifying the conditions under which time autonomy turns from a resource into a demand (Bakker and Demerouti 2017). When digital collaboration is low, high time autonomy appears to buffer the effect of time pressure on spillover, thereby preventing exhaustion. This finding supports research that perceives time autonomy, e.g., in the form of flextime, to have many benefits, such as better work-life balance and less work-family conflict (Allen et al. 2013). However, when both time autonomy and digital collaboration are high our data indicate that time autonomy turns from a resource into a demand and may even increase the effect of time pressure on spillover, leading to higher exhaustion. Work interdependencies between employees can affect productivity as an employee has to wait for inputs or replies. This may be why employees seek to meet deadlines even better (Morrison-Smith and Ruiz 2020) and accept the cost of work interfering with their personal lives (Baeriswyl et al. 2018) when they need to collaborate digitally and have the autonomy to work longer hours to complete their work (Lott 2018). Reasons for these effects may be, that high job design demands may lead employees to experience high responsibility to get their work done in a self-organized way. This may result in self-endangering behavior such as not taking breaks or working longer hours (Wendsche and Lohmann-Haislah 2016; Wong et al. 2019). In settings with intense digital collaboration this may be even more important, as employee expectations and mental health may be more difficult to perceive through virtual communication. The responsibility employees experience in this scenario may further result in rumination, such that spillover prevents employees from relaxation which would be necessary to reduce exhaustion (Kinnunen et al. 2019). In principle, the combination of high time autonomy and digital collaboration offers unlimited opportunities for interested self-endangerment, since compliance to a maximum limit of working hours per day and effects on mental health can remain largely unobserved.

### Practical implications: thoughtful leadership and self-leadership

This study shows that job design is very important, as modern working arrangements can lead to different outcomes depending on how they are designed and adapted to the needs of employees. Spillover from work to private life can be the result of poorly delegated tasks and set deadlines. Therefore, leaders should reflect the effect of time pressure. If leaders exert a lot of time pressure to get their employees to do their work on time, they should consider that it may result in spillover. To promote a productive and healthy work environment, they should regularly check to see if spillover is a problem. For example, by asking affected employees or themselves. If this is the case, they should consider reducing the time pressure to build up resources before increasing time pressure again. This reduction of time pressure should be analyzed individually for each person and situation as job design is complex (Mütze-Niewöhner et al. 2021). A reduction of time pressure might, for example, be achieved by working out a realistic plan with one’s colleagues or by using one’s social resources, such as asking colleagues for help, who have less pressure or have more task experience. Both can also be done in virtual meetings. Furthermore, joint team agreements regarding availability or respective company regulations could limit spillover from work into private life. It is crucial to keep in mind, that organizational structures and leaders can be key to achieving real change, as they often define the goals for individuals and teams (Neumer and Nicklich 2021). Leaders as well as employees can both be responsible for acting thoughtfully. Spillover from work to private life is particularly likely in situations where there is high time pressure combined with high time autonomy and a high degree of digital collaboration. When employees find themselves in this situation, they might change one of the three factors in order to deal with the demanding situation. One example of dealing with digital collaboration demands might be to set aside a few hours in one’s calendar for undisturbed work. Therefore, we recommend leaders and self-leaders to be thoughtful in their decisions, keeping possible unwanted outcomes in mind. As the analyzed scenario of high time pressure, time autonomy and digital collaboration can be linked to job design demands, the development of individual job design competencies could help. Dettmers and Clauß (2018) recommend building competences for planning and structuring one’s own work tasks, regulating work demand, the quantity and quality of work results, developing strategies to motivate oneself, designing one’s social environment, regulating one’s boundary between work and private life and planning of (work-)free recreation time. In addition, high work design demands require active reduction of stress at work and general health competence (Dettmers and Clauß 2018, p. 13). Organizations could support this competence development by supplying resources like time or information. Further, leaders should be aware that employees who work from home may feel socially isolated. Wang et al. (2021) identified the feeling of loneliness as an important challenge among remote workers during the pandemic. Their study shows that online social interactions are not necessarily sufficient for reducing loneliness. They explain this effect by restricted “intimacy” and “closeness”, despite real-time social interactions enabled by modern communication technology. Social isolation is positively related to psychological strain and negatively to job satisfaction (Bentley et al. 2016) and to organizational identification (Bartel et al. 2012). Therefore, organizations should offer employees to also work on site and to have face-to-face meetings besides offering them to work from home, if there are no pandemic restrictions.

### Limitations and directions for future research

There are some limitations to our study. Generalizability may be limited, as most respondents were studying part-time at a university, *besides working almost full-time in a company in average. Therefore, we can’t be sure, whether they interpret questions regarding their “work” only with respect to their job. If respondents referred the term work to both their job and their studies this might lead to higher time pressure due to working almost full time and studying on top compared to employees who do not study besides their job. However, the focus of our study is exactly on the negative effects of time autonomy when people work under high time pressure. If the sample were so homogenous by the double workload of work and study the chance of finding the expected effects would be reduced due to restricted variance, and our results would rather underestimate than overestimate existing relationships. To increase external validity of our findings, future studies could focus on other samples characterized by an even higher range of time pressure, time autonomy and digital collaboration.* Since this is cross-sectional data, no definite causal conclusions can be drawn from the study. All data is self-reported, hence common method bias might be an issue. However, common method bias does not seem to be a plausible alternative explanation for the reported moderation effects, as common method bias reduces test power, and the effect size estimates for a complex moderated relationship. Thus, “finding significant interaction effects despite the influence of common method bias in the data set should be taken as strong evidence that an interaction effect exists” (Siemsen et al. 2010, p. 470). Nevertheless, multisource data and a longitudinal design would promote further research. Longitudinal studies seem also warranted as effects might differ over time (O’Laughlin et al. 2018). Another limitation may be the time at which data was inquired: It was at the beginning of the pandemic when disrupt changes in digital collaboration overwhelmed organizations. Future research could therefore try to replicate the study. Further, research could focus on opportunities to reduce the demands by focusing on job design competencies (Dettmers and Bredehöft 2020). This accounts for leaders as well as employees themselves. For example, an experiment with a training in self-leadership strategies as an intervention could lead to further insight into coping mechanisms in the context of high work demands. Last but not least, our sample size was quite small, which reduced the likelihood to find interaction effects and might limit replicability of results. As research has shown that various design, measurement, and statistical artifacts bias observed moderating effects downwardly, so that average effect sizes of interaction effects are very small (Aguinis et al. 2005), we decided to use a 90% confidence interval (*p* < 0.10) for hypotheses testing, despite that we postulated directional effects in our hypotheses, that would allow for one-sided hypotheses testing, making it more probable to find significant effects. Given the small sample size and the actually low probability to detect an interaction effect, the error of rejecting an existing interaction effect seemed more probable than accepting a non-existent interaction effect. Anyway, recent statistical research strongly argues that *p* values should not be taken as thresholds but rather treated continuously and used as one among many pieces of evidence (McShane et al. 2019).

## Conclusion

To prevent that time pressure increases exhaustion via spillover from work to private life, leaders and employees should handle time pressure responsibly. Particularly, when employees work remotely and interdependenty, time autonomy and digital collaboration are crucial job-design demands and require mindful consideration as they can buffer or increase the effect of time pressure on exhaustion via on spillover. Joint team agreements and company regulations regarding availability and competences for coping with job design demands should be developed to limit spillover.

## Supplementary Information

**ESM 1 **Main and Interactive Effects of Time Pressure, Time Autonomy and Digital Collaboration on Job-to-Home Spillover

**ESM 2** Conditional Effects of Time Pressure on Job-to-Home Spillover at Values of the Moderator(s) Time Autonomy and Digital Collaboration

**ESM 3** Measurement of Items as Used in the Questionnaire
