# Supplementary material for: Negative effects of time autonomy in digital collaboration
Source: Gr Interakt Org. 2023 Feb 21;54(1):127–36. doi: 10.1007/s11612-023-00671-y (PMC9942660; doi:10.1007/s11612-023-00671-y)
Supplement: Supplementary file 1 — ESM 1 Main and Interactive Effects of Time Pressure, Time Autonomy and Digital Collaboration on Job-to-Home Spillover [file 11612_2023_671_MOESM1_ESM.docx]

**Electronic Supplementary Material 1**

**ESM 1** Main and interactive Effects of time pressure, time autonomy and digital collaboration on job-to-home spillover

|  |  |  | 90% CI | |
| --- | --- | --- | --- | --- |
| Variables | Coeff | *SE* | LL | UL |
| constant | -3,7905 | 2,7814 | -8,4070 | ,8260 |
| Time pressure | 2,5497 | ,9402 | ,9892 | 4,1102 |
| Time autonomy | 1,3721 | ,6846 | ,2358 | 2,5083 |
| Int_1 | -,5718 | ,2369 | -,9650 | -,1786 |
| Digital collaboration | ,1221 | ,0412 | ,0537 | ,1905 |
| Int_2 | -,0375 | ,0135 | -,0599 | -,0151 |
| Int_3 | -,0318 | ,0099 | -,0482 | -,0153 |
| Int_4 | ,0101 | ,0033 | ,0046 | ,0157 |

*Note. N* = 111; bootstrap sample size = 10,000. DV = dependent variable; 90 % CI = confidence interval; LL = lower limit; UL = upper limit. Int_1 = Time pressure x Time autonomy; Int_2: pressure x Digital collaboration; Int_3 = Time autonomy x Digital collaboration; Int_4 = Time pressure x Time autonomy x Digital collaboration
