# Supplementary material for: Negative effects of time autonomy in digital collaboration
Source: Gr Interakt Org. 2023 Feb 21;54(1):127–36. doi: 10.1007/s11612-023-00671-y (PMC9942660; doi:10.1007/s11612-023-00671-y)
Supplement: Supplementary file 2 — ESM 2 Conditional Effects of Time Pressure on Job-to-Home Spillover at Values of the Moderator(s) Time Autonomy and Digital Collaboration [file 11612_2023_671_MOESM2_ESM.docx]

**Electronic Supplementary Material 2**

**ESM 2** Conditional effects of time pressure on job-to-home spillover at values of the moderator(s) time autonomy and digital collaboration

|  |  |  |  | 90% CI | |
| --- | --- | --- | --- | --- | --- |
| Time  autonomy | Digital collaboration | Effect  Job-to-home spillover | *SE* | LL | UL |
| 2.9400 | 30.5923 | .6301 | .1759 | .3381 | .9220 |
| 2.9400 | 61.8018 | .3867 | .1037 | .2146 | .5588 |
| 2.9400 | 93.0113 | .1433 | .1677 | -.1350 | .4217 |
| 3.8000 | 30.5923 | .4043 | .1189 | .2069 | .6017 |
| 3.8000 | 61.8018 | .4323 | .0754 | .3073 | .5574 |
| 3.8000 | 93.0113 | .4604 | .1129 | .2730 | .6477 |
| 4.6600 | 30.5923 | .1786 | .1687 | -.1014 | .4586 |
| 4.6600 | 61.8018 | .4780 | .1026 | .3077 | .6483 |
| 4.6600 | 93.0113 | .7774 | .1349 | .5534 | 1.0014 |

*Note. N* = 111; bootstrap sample size = 10,000. DV = dependent variable; 90% CI = confidence interval; LL = lower limit; UL = upper limit.
