# Supplementary material for: Negative effects of time autonomy in digital collaboration
Source: Gr Interakt Org. 2023 Feb 21;54(1):127–36. doi: 10.1007/s11612-023-00671-y (PMC9942660; doi:10.1007/s11612-023-00671-y)
Supplement: Supplementary file 3 — ESM 3 Measurement of Items as Used in the Questionnaire [file 11612_2023_671_MOESM3_ESM.docx]

**ESM 3** Measurement of Items as Used in the Questionnaire

| **Variables** | **Measurement (German)** | **Measurement (English Translation)** |
| --- | --- | --- |
| Time Pressure | Bitte geben Sie an, wie gut die folgenden Aussagen Ihre Arbeit in den letzten vier Wochen beschreiben. ("stimme gar nicht zu" bis "stimme voll zu") | Please indicate how well the following statements describe your work over the past four weeks. ("strongly disagree" to "strongly agree") |
|  | Ich stehe häufig unter Zeitdruck. | I am often under time pressure. |
|  | Ich habe zu viel Arbeit. | I have too much work. |
|  | Ich muss oft schneller arbeiten, als ich es normalerweise tue, um meine Arbeit zu schaffen. | I often have to work faster than I normally do to get my work done. |
|  | Wegen der vielen Arbeit mache ich oft keine Pause oder später als geplant. | Because of all the work, I often don't take a break or I take a break later than planned. |
|  | Wegen zuviel Arbeit mache ich oft meinen Feierabend später als geplant. | Because of too much work, I often finish later than planned. |
|  |  |  |
| Time Autonomy | Bitte geben Sie an, wie gut die folgenden Aussagen Ihre Arbeit in den letzten vier Wochen beschreiben. ("stimme gar nicht zu" bis "stimme voll zu") | Please indicate how well the following statements describe your work over the past four weeks. ("strongly disagree" to "strongly agree") |
|  | Ich kann mein Arbeitstempo selbst bestimmen. | I can determine my own pace of work. |
|  | Ich kann selbst bestimmen, wann ich eine Pause mache. | I can decide for myself when to take a break. |
|  | Ich kann die Termine bei meiner Arbeit selbst planen. | I can schedule the appointments at my own work. |
|  | Ich kann bei meiner Arbeit mitentscheiden, wann ich was mache. | I have a say in when I do what in my work. |
|  | Ich kann selbst entscheiden, welcher Arbeitstätigkeit ich wann nachgehe. | I can decide for myself what work activity I pursue when. |
|  |  |  |
| Job-to-Home Spillover | Wie stark immen Sie den Aussagen zu, wenn Sie die Zeit seit den letzten vier Wochen rückblickend betrachten? | How strongly do you agree with the statements when you look back over the time since the last four weeks? looking back? |
|  | Ich hatte wegen meiner Arbeit nicht genug Zeit für mich selbst. | I didn't have enough time for myself because of my work. |
|  | Ich hatte wegen meiner Arbeit nicht genug Zeit für meine Familie oder andere wichtige Personen in meinem Leben. | I didn't have enough time for my family or other important people in my life because of my work. |
|  | Ich hatte wegen meiner Arbeit nicht genug Energie, um etwas mit meiner Familie oder mir wichtigen Personen zu unternehmen. | I didn't have enough energy to do anything with my family or people important to me because of my work. |
|  | Ich war wegen meiner Arbeit nicht jeden Tag in der Lage allen privaten Verpflichtungen nachzukommen. | I was not able to get everything done at home each day because of my work. |
|  | Ich war wegen meiner Arbeit nicht so gut gelaunt als ich zu Hause war. | I was not in such a good mood when I was at home because of my work. |
|  |  |  |
| Emotional Exhaustion | Bitte geben Sie an, inwieweit die folgenden Aussagen zutreffen. ("stimme gar nicht zu" bis "stimme voll zu") | Please indicate to what extent the following statements apply. ("strongly disagree" to "strongly agree") |
|  | Ich fühle mich durch meine Arbeit emotional erschöpft. | I feel emotionally exhausted by my work. |
|  | Am Ende eines Arbeitstages fühle ich mich verbraucht. | At the end of a workday, I feel used. |
|  | Ich fühle mich müde, wenn ich morgens aufstehe und den nächsten Arbeitstag vor mir habe. | I feel tired when I get up in the morning and have the next day of work ahead of me. |
|  | Den ganzen Tag zu arbeiten ist für mich wirklich anstrengend. | Working all day is really exhausting for me. |
|  | Ich fühle mich durch meine Arbeit ausgebrannt. | I feel burned out by my work. |
|  | Für mich ist die Arbeit auf Dauer nicht durchzuhalten. | For me, the work is not sustainable in the long run. |
| Digital Collaboration | Bitte ziehen Sie den Schieberegler an die entsprechende Prozentzahl. | Please drag the slider to the appropriate percentage. |
|  | Wie häufig arbeiten Sie mit anderen Personen über digitale Medien zusammen? ("nie"=0% bis "immer"=100%) | How often do you collaborate with others via digital media? ("never"=0% to "always"=100%) |
